# Endocan: A Novel Marker for Colchicine Resistance in Familial Mediterranean Fever Patients?

**DOI:** 10.3389/fped.2021.788864

**Published:** 2021-11-29

**Authors:** Ahmet Omma, Berkan Armaǧan, Serdar Can Güven, Sevinç Can Sandıkçı, Seda Çolak, Çiǧdem Yücel, Orhan Küçükşahin, Abdulsamet Erden

**Affiliations:** ^1^Department of Rheumatology, Ankara Numune Training and Research Hospital, Ankara, Turkey; ^2^Clinic of Rheumatology, Ankara City Hospital, Ankara, Turkey; ^3^Department of Clinical Biochemistry, Ankara Numune Training and Research Hospital, Ankara, Turkey; ^4^Division of Rheumatology, Department of Internal Medicine, Ankara City Hospital, Yildirim Beyazit University, Ankara, Turkey

**Keywords:** endocan, FMF attacks, novel biomarker, colchicine responsiveness, colchicine resistance, FMF

## Abstract

**Introduction:** Familial Mediterranean fever (FMF) patients had 5–10% colchicine resistance. Although FMF attacks are characterized by acute phase elevation, there are no biomarkers that can show colchicine resistance yet. The serum endocan levels may elevate in inflammatory and auto-inflammatory diseases.

**Objectives:** This study aimed to evaluate serum endocan levels in FMF patients according to whether attack and colchicine resistance or not and also compare them with classical acute phase reactants.

**Methods:** In this single-center and cross-sectional study, a total of 111 FMF patients and 60 healthy individuals were enrolled. All patients' basic demographic and clinical data were recorded and blood samples were collected.

**Results:** A total of 46 (41.4%) FMF patients had colchicine resistance. In comparison to the FMF patients according to colchicine response, colchicine resistance patients had a significantly higher median (IQR) endocan levels than colchicine responsive patients [36.98 ng/ml (97.41) vs. 13.57 ng/ml (27.87), *p* = 0.007], but there were no differences between in terms of median ESR and CRP levels. Inversely, serum endocan levels were similar during an attack and attack-free period in FMF patients, although ESR and CRP levels were significantly different. Interestingly, the highest serum endocan levels were in the control group.

**Conclusion:** In conclusion, serum endocan levels were higher in colchicine resistance than colchicine responsive patients, but attack state had no effect on serum endocan levels in our study. Unlike ESR and CRP, serum endocan may be a novel biomarker for detection of colchicine resistance and distinguish the FMF attacks.

## Introduction

Endocan, formerly known as endothelial cell-specific molecule 1, is a novel inflammatory marker which is secreted from vascular endothelium in addition to endothelial cells of various organs and plays a key regulatory role during the course of inflammatory diseases and neoplasms (1, 2). Pro-inflammatory cytokines such as tumor necrosis factor alpha (TNF-α) and interleukin 1-beta (IL1-B) had been demonstrated to induce endocan production *in vitro*, while interferon gamma (IFN-γ) had been demonstrated to inhibit. Accordingly, serum endocan levels were revealed to elevate in inflammatory processes such as infections, sepsis, malignancies, and auto-inflammatory diseases (3). Familial Mediterranean fever (FMF) is a hereditary auto-inflammatory disease characterized by recurrent febrile polyserositis attacks (4). Colchicine is the mainstay therapy for FMF which effectively prevents both inflammatory attacks and chronic inflammation, as well as development of secondary amyloidosis (5). However, ~5–10% of FMF patients can be resistant to colchicine therapy (6). Despite accumulation of knowledge to a degree in the current literature, the reason for colchicine resistance in FMF patients still remains obscure. Chronic inflammation in inadequate or partial FMF treatment leads to comorbidities such as secondary amyloidosis, chronic renal failure, and cardiovascular diseases. Impaired endothelial dysfunction has been responsible for these FMF complications and could emerge before organ damages (7, 8).

In this study, we aimed to evaluate serum endocan levels in FMF patients according to whether FMF attack and colchicine resistance or not and also compare them with classical acute phase reactants.

## Methods

### Study Design and Patients' Selection

In this single-center, cross-sectional study, 111 FMF patients who consecutively referred to the adult rheumatology outpatient clinics of Ankara Numune Training and Research Hospital were enrolled between January and December 2018.

All of the FMF patients met the Tel-Hashomer criteria (9). A total of 60 healthy individuals who were admitted to our rheumatology clinic without any eventual diagnosis for any rheumatic condition or comorbid diseases were included as the control group. All of the healthy individuals' acute phase reactants levels were normal. Tobacco and alcohol consumers, FMF patients under any medication other than colchicine, and patients who were pregnant or in postpartum period (up to 6 months) were also excluded.

### FMF Assessment and Colchicine Resistance

Demographics, clinical features, laboratory findings, number of attacks per year before and after colchicine treatment, presence of IL-1 blocker use and the reason for IL-1 blocker initiation, and Mediterranean fever (MEFV) variant analysis were recorded by medical file screening and face-to-face interview. Imported colchicine preparations are used before biological treatments in case of unresponsiveness to local colchicine preparations in our country in FMF patients. In the FMF patients who are unresponsive to import colchicine treatment, IL-1 blocker (for example, anakinra and canakinumab) treatments are used in our country (10). MEFV mutation analysis was studied in 66% of the patients. Investigators (AO, SÇ, SCS) performed the physical examinations of all patients. Response to colchicine was also assessed by the same investigators using questionnaires; colchicine unresponsiveness or resistance (Group CResistant) was defined as more than three typical FMF attacks within the last 6 months despite treatment with at least 2 mg of colchicine daily, and patients who had none of this criteria were grouped as colchicine responsive patients (Group CResponsive) (11). Briefly, the criteria defining FMF attack included all of the following: fever (≥38°C) lasting from 6 to 72 h accompanied by painful clinical findings of serositis/arthritis, skin rash and elevated C-reactive protein (CRP; mg/L, normal value ≤5), and/or erythrocyte sedimentation rate (ESR; mm/hour, normal range 0–20) levels.

### Blood Sampling

Venous blood samples were collected from the three groups after 8–12 h of fasting upon written consent for participation was given. Blood samples collected for analysis were centrifuged at 4,000 rpm for 10 min. Separated sera were aliquoted into Eppendorf tubes and stored at −80°C until the time of analysis. Serum endocan levels were detected with human *enzyme linked immunosorbent assay* (ELISA, double antibody sandwich method) kits according to the manufacturer's instructions (Cloud Clone Corp, TX, USA). Values were expressed in ng/ml for endocan.

### Statistical Analyses

Statistical analysis was performed using the Statistical Package for the Social Sciences software (version 21.0; IBM Corporation, Armonk, NY, USA). The variables were investigated using visual (histogram, probability plots) and analytic methods (Kolmogorov-Smirnov) to determine whether or not they are normally distributed. The data of descriptive analysis were expressed as the median and interquartile range (IQR) values. Categorical variables were compared with the chi-square test or Fisher's exact test where appropriate. The Mann-Whitney *U* test was used to compare the non-normally distributed continuous data between two groups. *p* < 0.05 was considered as statistically significant.

## Results

### Demographics and Clinical Features

The study sample consisted of 111 FMF patients and 60 healthy individuals. Mean (SD) age for FMF patients was 36.3 (11.4) and for healthy controls was 36.1 (9.8) without significant difference (*p* = 0.909). A total of 55 (49.5%) patients were male in the FMF group and 30 (50%) were male in the control group (*p* = 0.955).

A total of 46 (41.4%) FMF patients were CResistant. Blood samples were taken during an FMF attack in 34 (30.6%) of the patients. Of the 46 CResistant patients, 24 (52.2%) were treated with anakinra, 3 (6.5%) with canakinumab, and 19 (41.3%) with imported colchicine. Demographics and clinical features of FMF patients were presented in Table 1. Median (min-max) age for symptom onset was 8 years (2–48) in CResistant and was 20 years (5–59) in CResponsive (*p* < 0.0001). Median (min-max) age at diagnosis was 20.5 (5–60) years in CResistant group and 30 (7–60) years in CResponsive group (*p* = 0.003). Clinical manifestations of attacks were similar between CResistant and CResponsive groups except for arthritis which was more frequent in the CResistant group [*n* = 25 (54.3%) vs. *n* = 20 (30.8%), *p* = 0.013]. In comparison to the CResponsive group, the CResistant group had a higher frequency of amyloidosis and end stage renal disease (30.4% vs. 0, *p* < 0.0001 and 15.2% vs. 0, *p* = 0.002, respectively).

**Table 1:** The demographics and clinical characteristics of familial Mediterranean fever patients.

	**All patients (*n* = 111)**	**CResistant group (*n* = 46)**	**CResponsive group (*n* = 65)**	***p***
Age, years, median (IQR)	37 (15)	37.5 (17)	36 (14)	0.43
Age at symptom onset, years, median (IQR)	16 (19)	8 (10)	20 (18)	**<0.0001**
Age of diagnosis, years, median (IQR)	26 (18)	20.5 (21)	30 (15)	**0.003**
Follow duration, months, median (IQR)	90 (136)	160 (162)	70 (92)	**<0.0001**
Female, *n* (%)	86 (50.3)	24 (52.2)	32 (49.2)	0.76
Abdominal pain, *n* (%)	105 (94.6)	43 (93.5)	62 (95.4)	0.662
Fever, *n* (%)	102 (91.9)	43 (93.5)	59 (90.8)	0.606
Arthritis, *n* (%)	45 (40.5)	25 (54.3)	20 (30.8)	**0.013**
Pleuritic chest pain, *n* (%)	41 (36.9)	19 (41.3)	22 (33.8)	0.423
Pericarditis, *n* (%)	0 (0)	0 (0)	0 (0)	-
Protracted febrile myalgia, *n* (%)	6 (5.4)	3 (6.5)	3 (4.6)	0.691
Vasculitis, *n* (%)	7 (6.3)	5 (10.9)	2 (3.1)	0.124
Henoch-Schonlein purpura, *n* (%)	5 (4.5)	3 (6.5)	2 (3.1)	
Polyarteritis nodosa, *n* (%)	2 (1.8)	2 (4.3)	0 (0)	
Erysipelas-like erythema, *n* (%)	16 (14.4)	9 (19.6)	7 (10.8)	0.194
Amyloidosis, *n* (%)	14 (12.6)	14 (30.4)	0 (0)	**<0.0001**
End stage renal disease, *n* (%)	7 (6.3)	7 (15.2)	0 (0)	**0.002**
Family history of FMF^a^, *n* (%)	59 (53.2)	25 (54.3)	34 (52.3)	0.906
ESR (mm/h), median (IQR)	12 (18)	17.5 (2–78)	11 (1–46)	0.931
CRP (mg/L), median (IQR)	7 (22)	7 (26)	7 (19)	0.083
Carriers of M694V/M694V, *n* (%)	20 (18)	13 (28.3)	7 (10.8)	**0.018**

*CResistant, Colchicine unresponsive; CResponsive, Colchicine responsive; CRP, C-reactive protein; ESR, erythrocyte sedimentation rate; FMF, familial Mediterranean fever; SD, standard deviation*.

a *Family history of FMF was known in only 59 patients. P-values marked with bold indicate statistically significant differences between the groups*.

### Endocan and Colchicine Response

**Figure 1:**
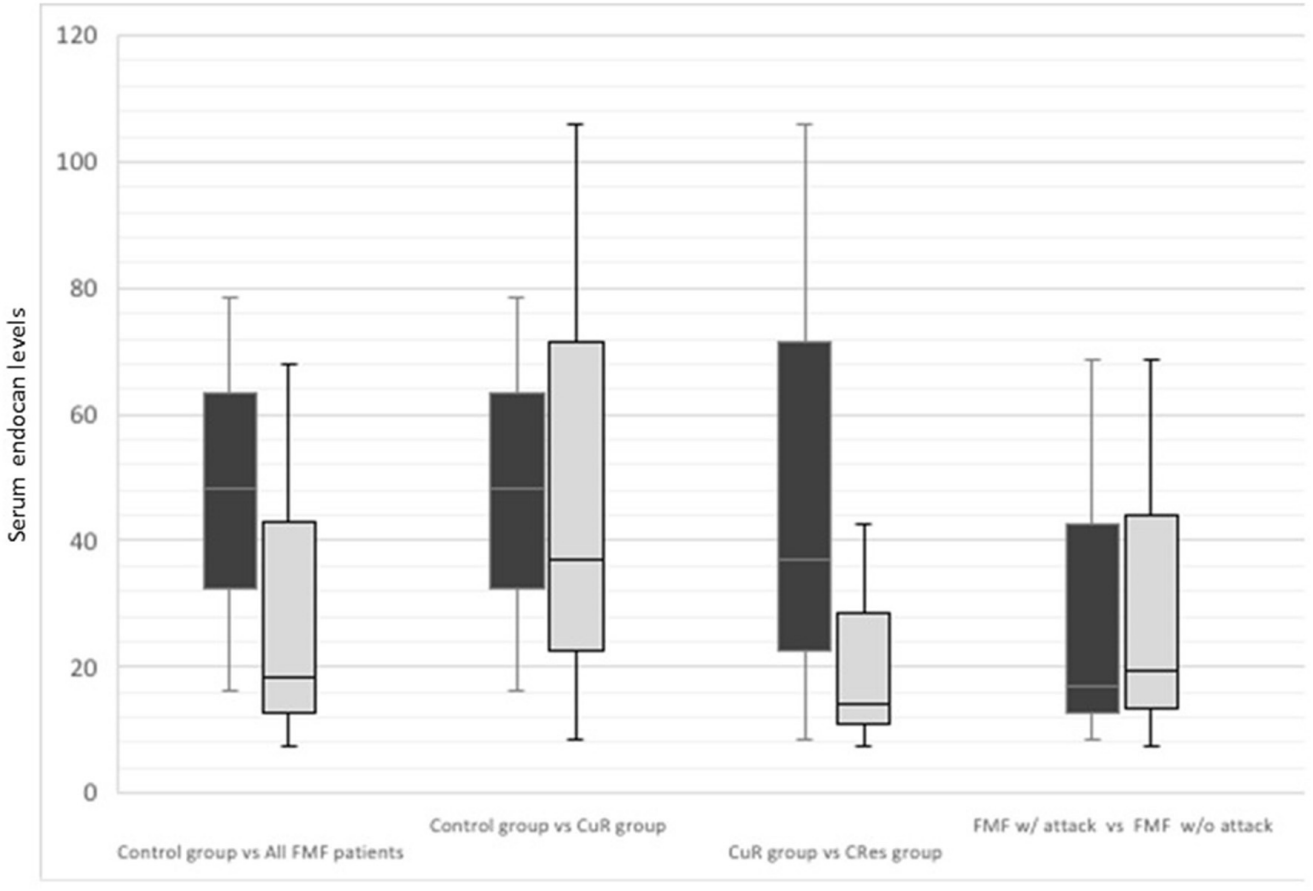
The comparison of the endocan levels among control group and all familial Mediterranean fever patients' subgroups.

The comparison of the endocan levels among control and FMF groups was presented in Figure 1. The median (IQR) endocan levels were lower in all FMF patient groups than the control group [17.52 ng/ml (53.06) vs. 48.35 ng/ml (62.22), *p* = 0.005]. But in comparison to the CResistant group, the control group had slightly higher endocan levels without statistical significance (*p* = 0.726). When we compared the FMF patients according to colchicine response, CResistant group had a significantly higher median (IQR) endocan levels than CResponsive group [36.98 ng/ml (97.41) vs. 13.57 ng/ml (27.87), *p* = 0.007], but there were no differences between in terms of median ESR and CRP levels (*p* = 0.931 and *p* = 0.083, respectively) (Table 1). Serum endocan levels were similar during an attack and attack-free period in FMF patients. FMF patients with attack had a higher frequency of a median (IQR) ESR (mm/h) and CRP (mg/L) levels than attack-free patients [22 (24) vs. 9 (13), *p* < 0.001 and 40.5 (71) vs. 3 (8), *p* < 0.001, respectively]. When we compare the MEFV mutation groups as no mutation (25%), homozygous (16%), heterozygous (11%), and compound heterozygous (14%), there were no significant differences among the groups.

## Discussion

Our results showed that, in FMF patients, the CResistant group had significantly higher serum endocan levels than the CResponsive group. Furthermore, endocan levels did not differ between attacks and attack-free periods. Acute phase reactants (ESR, CRP) were higher in patients with an attack; however, their levels were similar between CResponsive and CResistant groups. Interestingly, the highest serum endocan levels were observed in the control group in contrast to the FMF patients.

Colchicine is a potent inhibitor of IL-1 pathway and neutrophil activation *via* microtubule inhibition (12). In addition to being the mainstay treatment in FMF, response to colchicine is also the most important predictor of long-term outcome (13). Unresponsiveness to colchicine could affect the quality of life with frequent and severe attacks and even increase the possibility of secondary amyloidosis. Therefore, CResistant patients have been under an increased risk of mortality from amyloidosis or developing other conditions related to chronic inflammation (13). IL-1 receptor antagonists have emerged as effective treatment options for suppressing auto-inflammation in colchicine resistant patients (14). Accordingly, early detection of FMF patients with inadequate response to colchicine and initiating an alternative treatment is crucial. Patients may seldom have only acute phase elevation without clinical complaints. Less frequently, patients may present with AA amyloidosis without any preceding clinical complaints or detected acute phase elevation, so-called “phenotype 2.” Various biomarkers are generally evaluated during follow-ups in FMF patients, mainly ESR and CRP, to monitor disease activity and treatment adequacy. The subclinical inflammation may continue during attack-free periods in FMF patients, and despite reflecting attacks with high sensitivity, value of the classical acute phase reactants (ESR and CRP) for the prediction of subclinical inflammation may progress to AA amyloidosis (15, 16). In addition, in about 30% of patients with FMF, the laboratory markers of inflammation remain elevated even after an attack resolves (13–19). The evidence supporting the monitoring of FMF with any particular acute phase reactant, including serum amyloid A, over the others is limited (16). At this point, it seems to be a necessity to define a marker that may represent inadequate treatment better than classical acute phase reactants in FMF. In our study, endocan levels were higher in the CResistant group despite no significant differences in CRP and ESR levels, which may be speculated that endocan may be an option to detect colchicine resistance.

Major function of endocan is regulating leukocyte transmigration and adhesion to endothelial *via* taking part in lymphocyte function-associated antigen 1 (LFA-1) and intracellular adhesion molecule 1 (ICAM-1) integration. During inflammation, endocan plays an inhibitory role in leukocyte adhesion, migration, and eventually extravasation by binding LFA-1 and blocking its integration to ligands on intravascular endothelium (20). In various studies, which included chronic inflammatory rheumatic diseases other than FMF, serum endocan levels had been correlated with disease activity (21–23). Else, serum endocan level was more useful than acute phase reactants in some infectious diseases such as pneumonia (24, 25). However, endocan levels did not seem to elevate during attacks in our study. Since endocan release from inflammatory cells is not a fast process, it is possible that endocan may be more useful to detect a long ongoing, chronic inflammatory state, rather than rapid and brief hyper-inflammatory reactions like an FMF attack.

The exact reasons for inadequate response to colchicine are yet to be fully clarified. Many factors such as ethnicity, genetic mutations, varying colchicine metabolism, gastrointestinal absorption, and insufficiently low prescribed colchicine doses may be responsible (13, 26–30). In a study, M694V/M694V homozygous mutation is associated with lower response to colchicine treatment (31). Chronic musculoskeletal involvement is usually resistant to colchicine (29). Lidar et al. defined clinical features of CResistant. These patients had more severe disease and more prevalent abdominal, pleural, articular, and scrotal attacks and erysipeloid eruptions (27). On the other hand, Erden et al. found that the frequency of the arthralgia, arthritis, pleuritic chest pain, and erysipelas-like erythema was higher in the CResistant group than CResponsive group (26). In our study, the only significant difference between the CResistant and CResponsive group was that arthritis was more common in the CResistant group. According to these data, it should be kept in mind that colchicine resistance is high in FMF patients with articular involvement.

Effective inhibition of inflammation and neutrophil activity by colchicine treatment may be related with downregulation of endocan secretion (12). Due to the anti-inflammatory effects of colchicine, which had been demonstrated in previous studies, serum endocan levels may be detected significantly higher in healthy controls compared to all FMF patients and CResponsive groups in our study. Although the CResistant group had a significantly higher serum endocan levels than the CResponsive group, there was no difference when compared to healthy controls. In this study, in which all FMF patients received colchicine, the lower endocan levels in the CResponsive group compared to the healthy controls and the CResistant group may again be related to colchicine's effect on suppressing inflammation and may be representative of treatment adequacy. In addition, serum levels may be affected by some medications. Lipid-lowering therapy and some antihypertensive drugs may lower endocan levels (32, 33). Different from healthy controls who had no comorbidity, our FMF patients also had some comorbidities. In this context, other drugs except colchicine may also be responsible for this difference.

The major limitations of our study were the small sample size in the subgroups and the single-centered, retrospective nature of the study. Drug compliance was determined according to patients' statements and pill counts. MEFV mutations were not screened in healthy controls. Thus, the real frequency of carriers among healthy patients was not known. For a better comparison of the results, a standardized severity scoring system for FMF was needed instead of just ESR and CRP. Lastly, in order to make a sense interpretation of the relationship between the endocan levels and FMF attacks, endocan levels should be measured at the same patient on attack and attack-free periods.

In conclusion, serum endocan levels were higher in CResistant patients than CResponsive patients, but the attack state had no effect on serum endocan levels in our study. Under colchicine treatment, endocan levels may not distinguish FMF attacks; however, it may be a convenient marker to evaluate colchicine resistance. According to our incidental finding, colchicine treatment, regardless of adequacy, had a lowering effect on serum endocan levels. Although these findings suggest that colchicine has a positive effect that increases serum endocan levels, presumably endothelial dysfunction mostly, further studies are needed to prove that.

## Data Availability Statement

The raw data supporting the conclusions of this article will be made available by the authors, without undue reservation.

## Ethics Statement

The studies involving human participants were reviewed and approved by Ankara Numune Training and Research Hospital of Ethics Committee (E-16-1072). All subjects gave written informed consent in accordance with the Declaration of Helsinki.

## Author Contributions

AO, SS, SÇ, and ÇY designed the study. ÇY, SS, and SÇ examined cases and collected data. AE, OK, BA, and SG searched the literature. AO, BA, SG, OK, and AE interpreted and discussed the results. AO, BA, and AE analyzed data. BA, OK, AE, SG, and AO wrote the paper. AO, BA, AE, OK, and SG revised the manuscript critically for important intellectual content and contributed to the final version of the manuscript. All authors have reviewed and approved the final version of the article, including the authorship list.

## Funding

This work was supported by the Ankara Numune Training and Research Hospital scientific research support fund (Grant No: 2016-1200).

## Conflict of Interest

The authors declare that the research was conducted in the absence of any commercial or financial relationships that could be construed as a potential conflict of interest.

## Publisher's Note

All claims expressed in this article are solely those of the authors and do not necessarily represent those of their affiliated organizations, or those of the publisher, the editors and the reviewers. Any product that may be evaluated in this article, or claim that may be made by its manufacturer, is not guaranteed or endorsed by the publisher.

## References

[1] Sarrazin S Adam E Lyon M Depontieu F Motte V Landolfi C. Endocan or endothelial cell specific molecule-1 (ESM-1): a potential novel endothelial cell marker and a new target for cancer therapy. Biochim Biophys Acta. (2006) 1765:25–37. 10.1016/j.bbcan.2005.08.004 16168566

[2] Zhang SM Zuo L Zhou Q Gui SY Shi R Wu Q. Expression and distribution of endocan in human tissues. Biotech Histochem. (2012) 87:172–8. 10.3109/10520295.2011.577754 21526908

[3] Brevetti G Schiano V Chiariello M. Cellular adhesion molecules and peripheral arterial disease. Vasc Med. (2006) 11:39–47. 10.1191/1358863x06vm645ra 16669412

[4] Ozen S Batu ED. The myths we believed in familial Mediterranean fever: what have we learned in the past years? Semin Immunopathol. (2015) 37:363–9. 10.1007/s00281-015-0484-6 25832989

[5] Livneh A Zemer D Langevitz P Shemer J Sohar E Pras M. Colchicine in the treatment of AA and AL amyloidosis. Semin Arthritis Rheum. (1993) 23:206–14. 10.1016/S0049-0172(05)80042-3 8122124

[6] Gul A. Treatment of familial Mediterranean fever: colchicine and beyond. Isr Med Assoc J. (2014) 16:281–4. 24979831

[7] Gunes H Kivrak T Tatlisu M Kaya H Yilmaz MB. Relationship between endothelial dysfunction and microalbuminuria in familial Mediterranean fever. Eur J Rheumatol. (2016) 3:61–4. 10.5152/eurjrheum.2016.15079 27708973 PMC5042232

[8] Yilmaz MI Demirkaya E Acikel C Saldir M Akar S Cayci T. Endothelial function in patients with familial Mediterranean fever-related amyloidosis and association with cardiovascular events. Rheumatology. (2014) 53:2002–8. 10.1093/rheumatology/keu231 24907154

[9] Livneh A Langevitz P Zemer D Zaks N Kees S Lidar T. Criteria for the diagnosis of familial Mediterranean fever. Arthritis Rheum. (1997) 40:1879–85. 10.1002/art.1780401023 9336425

[10] Emmungil H Ilgen U Turan S Yaman S Kucuksahin O. Different pharmaceutical preparations of colchicine for Familial Mediterranean Fever: are they the same? Rheumatol Int. (2020) 40:129–35. 10.1007/s00296-019-04432-3 31463607

[11] Erden A Batu ED Sari A Sonmez HE Armagan B Demir S. Which definition should be used to determine colchicine resistance among patients with familial Mediterranean fever? Clin Exp Rheumatol. (2018) 36:97–102. 30418112

[12] Karamanou M Tsoucalas G Pantos K Androutsos G. Isolating Colchicine in 19th century: an old drug revisited. Curr Pharm Des. (2018) 24:654–8. 10.2174/1381612824666180115105850 29336251

[13] Ben-Zvi I Livneh A. Chronic inflammation in FMF: markers, risk factors, outcomes and therapy. Nat Rev Rheumatol. (2011) 7:105–12. 10.1038/nrrheum.2010.181 21060333

[14] Kucuksahin O Yildizgoren MT Ilgen U Ates A Kinikli G Turgay M. Anti-interleukin-1 treatment in 26 patients with refractory familial mediterranean fever. Mod Rheumatol. (2017) 27:350–5. 10.1080/14397595.2016.1194510 27328763

[15] Duzova A Bakkaloglu A Besbas N Topaloglu R Ozen S Ozaltin F. Role of A-SAA in monitoring subclinical inflammation and in colchicine dosage in familial Mediterranean fever. Clin Exp Rheumatol. (2003) 21:509–14. 12942707

[16] Erer B Demirkaya E Ozen S Kallinich T. What is the best acute phase reactant for familial Mediterranean fever follow-up and its role in the prediction of complications? A systematic review. Rheumatol Int. (2016) 36:483–7. 10.1007/s00296-015-3413-z 26712372

[17] Berkun Y Padeh S Reichman B Zaks N Rabinovich E Lidar M. A single testing of serum amyloid a levels as a tool for diagnosis and treatment dilemmas in familial Mediterranean fever. Semin Arthritis Rheum. (2007) 37:182–8. 10.1016/j.semarthrit.2007.03.005 17512038

[18] Korkmaz C Ozdogan H Kasapcopur O Yazici H. Acute phase response in familial Mediterranean fever. Ann Rheum Dis. (2002) 61:79–81. 10.1136/ard.61.1.79 11779767 PMC1753891

[19] Lachmann HJ Sengul B Yavuzsen TU Booth DR Booth SE Bybee A. Clinical and subclinical inflammation in patients with familial Mediterranean fever and in heterozygous carriers of MEFV mutations. Rheumatology (Oxford). (2006) 45:746–50. 10.1093/rheumatology/kei279 16403826

[20] Bechard D Scherpereel A Hammad H Gentina T Tsicopoulos A Aumercier M. Human endothelial-cell specific molecule-1 binds directly to the integrin CD11a/CD18 (LFA-1) and blocks binding to intercellular adhesion molecule-1. J Immunol. (2001) 167:3099–106. 10.4049/jimmunol.167.6.3099 11544294

[21] Balanescu P Ladaru A Balanescu E Voiosu T Baicus C Dan GA. Endocan, novel potential biomarker for systemic sclerosis: results of a pilot study. J Clin Lab Anal. (2016) 30:368–73. 10.1002/jcla.21864 26331941 PMC6807019

[22] Balta I Balta S Demirkol S Celik T Ekiz O Cakar M. Aortic arterial stiffness is a moderate predictor of cardiovascular disease in patients with psoriasis vulgaris. Angiology. (2014) 65:74–8. 10.1177/0003319713485805 23636854

[23] Balta I Balta S Demirkol S Mikhailidis DP Celik T Akhan M. Elevated serum levels of endocan in patients with psoriasis vulgaris: correlations with cardiovascular risk and activity of disease. Br J Dermatol. (2013) 169:1066–70. 10.1111/bjd.12525 23889284

[24] Kao SJ Chuang CY Tang CH Lin CH Bien MY Yu MC. Plasma endothelial cell-specific molecule-1 (ESM-1) in management of community-acquired pneumonia. Clin Chem Lab Med. (2014) 52:445–51. 10.1515/cclm-2013-0638 24108208

[25] Kupeli I Salcan S Kuzucu M Kuyrukluyildiz U. Can endocan be a new biomarker in ventilator-associated pneumonia? Kaohsiung J Med Sci. (2018) 34:689–94. 10.1016/j.kjms.2018.07.002 30527203 PMC11915590

[26] Erden A Batu ED Armagan B Sonmez HE Sari A Demir S. Blood group 'A' may have a possible modifier effect on familial Mediterranean fever and blood group '0' may be associated with colchicine resistance. Biomark Med. (2018) 12:565–72. 10.2217/bmm-2017-0344 29873519

[27] Lidar M Scherrmann JM Shinar Y Chetrit A Niel E Gershoni-Baruch R. Colchicine nonresponsiveness in familial Mediterranean fever: clinical, genetic, pharmacokinetic, and socioeconomic characterization. Semin Arthritis Rheum. (2004) 33:273–82. 10.1053/S0049-0172(03)00137-9 14978665

[28] Ozer I Mete T Turkeli Sezer O Kolbasi Ozgen G Kucuk GO Kaya C. Association between colchicine resistance and vitamin D in familial Mediterranean fever. Ren Fail. (2015) 37:1122–5. 10.3109/0886022X.2015.1056064 26067744

[29] Rabinovich E Livneh A Langevitz P Brezniak N Shinar E Pras M. Severe disease in patients with rheumatoid arthritis carrying a mutation in the Mediterranean fever gene. Ann Rheum Dis. (2005) 64:1009–14. 10.1136/ard.2004.029447 15958759 PMC1755576

[30] Tufan A Babaoglu MO Akdogan A Yasar U Calguneri M Kalyoncu U. Association of drug transporter gene ABCB_1_ (MDR_1_) 3435C to T polymorphism with colchicine response in familial Mediterranean fever. J Rheumatol. (2007) 34:1540–4. 17610314

[31] Soylemezoglu O Arga M Fidan K. Unresponsiveness to colchicine therapy in patients with familial Mediterranean fever homozygous for the M694V mutation. J Rheumatol. (2010) 37:182–9. 10.3899/jrheum.090273 20008920

[32] Celik T Balta S Karaman M Ahmet Ay S Demirkol S Ozturk C. Endocan, a novel marker of endothelial dysfunction in patients with essential hypertension: comparative effects of amlodipine and valsartan. Blood Press. (2015) 24:55–60. 10.3109/08037051.2014.972816 25390761

[33] Tziomalos K Athyros VG Karagiannis A Mikhailidis DP. Lipid lowering agents and the endothelium: an update after 4 years. Curr Vasc Pharmacol. (2012) 10:33–41. 10.2174/157016112798829742 22112353

